# Evolving dynamic needs for patient-reported outcomes assessment in individuals with an abdominal aortic aneurysm (AAA): A systematic review

**DOI:** 10.1177/1358863X261417234

**Published:** 2026-03-19

**Authors:** Kim G Smolderen, Christiany Tapia, Bernard Dennis, Santiago Callegari, Marie Dahl, Jes S Lindholt, Isabelle Van Herzeele, Gaëlle Romain, Carlos Mena-Hurtado

**Affiliations:** 1 Department of Internal Medicine, Cardiovascular Medicine Section, Vascular Medicine Outcomes Lab, Yale School of Medicine, New Haven, CT, USA; 2 Department of Psychiatry, Psychology Section, Yale School of Medicine, New Haven, CT, USA; 3 Retired, Patient Advocate, USA; 4 Department of Vascular Surgery, Vascular Research Unit, Viborg Regional Hospital, Viborg, Denmark; 5 Department of Clinical Medicine, Aarhus University, Aarhus, Denmark; 6 Department of Cardiothoracic and Vascular Surgery, Odense University Hospital, Odense, Denmark; 7 Department of Thoracic and Vascular Surgery, Ghent University Hospital, Ghent, Belgium

**Keywords:** abdominal aortic aneurysm (AAA), flourishing, patient-reported outcomes, positive psychology

## Abstract

Abdominal aortic aneurysm (AAA) affects over 35 million individuals and poses a potentially fatal risk. Risk stratification and surveillance strategies are well established, but their impact on patient-reported outcomes (PROs) remains diffuse and may vary by clinical and biopsychosocial profiles. It is also unclear whether individuals with this often ‘silent’ condition experience thriving or growth concepts referred to as ‘flourishing.’ To address these gaps, we systematically reviewed PRO studies in AAA to answer three questions: (1) Are there instruments tailored to AAA phenotypes (rupture, elective repair, surveillance, screening), and what patient-centered dimensions are measured? (2) What mental health assessments are commonly used? (3) Do existing PROs capture elements of flourishing? We included 16 studies. Generic PRO tools were frequently used but limited in scope. Five AAA-specific instruments were identified, focusing on physical symptoms, treatment satisfaction, and disease burden. Most were developed using Classical Test Theory, with item burden ranging from 11 to 70 items, and primarily targeted surveillance or elective surgery populations. Mental health assessment was minimal – typically single items embedded in health status instruments addressing anxiety and depression. Flourishing was indirectly assessed through measures of social connection and emotional well-being. Current PRO approaches fail to capture the heterogeneous, patient-centered experience of living with AAA. We propose a growth-based framework for PRO evaluation using adaptive testing to accommodate clinical complexity while minimizing response burden, supporting integrative, value-based, patient-centered care.

## Introduction

Globally, abdominal aortic aneurysm (AAA) affects over 35 million individuals, with higher prevalence in Western nations due to risk factors such as smoking, male sex, older age, obesity, family history, and cardiovascular comorbidities.^1^ Upon rupture, less than 50% reach the operating table, more than one-third die with open repair, and at least one in five is offered endovascular repair.^2^ Screening is recommended in high-risk AAA groups (older men with smoking history),^3^ with demonstrated survival benefits.

AAA is often an asymptomatic condition until it progresses to critical, acute stages, and if one asks the patient what the experienced impacts are on multiple domains of functioning through patient-reported outcomes (PROs), not one size may fit all. The impact depends on the disease stage and timing of diagnosis.^4–6^ For example, one in three surgical repair patients may develop stress or trauma-related psychiatric conditions postsurgery.^7^ Screening may cause temporary stress, but overall depressive and anxiety symptoms may be similar to those in populations who do not undergo screening.^8–10^ On the other hand, patients who screen positive for AAA may experience impaired quality of life on a continued basis and progressive worsening as a result of psychosomatic distress during conservative management.^11^ Even though this population may present with heightened cardiovascular risk and a high burden of somatic comorbidities, a diagnosis of AAA may still be compatible with the overall well-being experienced within the developmental context of the natural progression of aging.^11^

Aligned with this view is the rise of integrated approaches in cardiovascular diseases that address the impact of chronic disease not only from the perspective of its physical impacts or longevity threats, but also by incorporating the mental and psychological impacts of disease and focusing on strategies to enhance well-being, even in the presence of chronic disease.^12^

AAA is no exception and is often more overlooked because it is believed to be asymptomatic in its early stages. However, the emotional toll is frequently not taken into account, yet it is very present and can prevent people from fully enjoying life and experiencing well-being, also referred to as ‘flourishing.’^13,14^ The idea of flourishing recognizes human capacities for positive emotions, social engagement, meaningful relationships, and a purposeful life.^15^ Even without mental health disorders, individuals may languish rather than thrive. Chronic illness does not prevent fulfilling lives, which current PRO assessments may overlook, missing a dimension of personal functioning. As people age and experience more health-related challenges, the importance of flourishing—encompassing competence, optimism, meaning, and emotional stability—becomes increasingly significant.^16^

This review aims to answer key questions about PRO measurement in AAA and to assess the extent to which a broadened approach to capturing aspects of flourishing is adopted. Specifically, (1) are there suitable PRO instruments tailored to clinical phenotype stages—such as rupture, repair, surveillance, and screening—and what do they measure? (2) Which mental health screenings and disorders are typically evaluated in patients with AAA? (3) Do current tools measure elements of flourishing?

The review will propose a multidimensional framework combining health status, quality of life, mental health, and well-being, alongside an adaptive testing methodology to support a holistic, precision-medicine approach to assessing PRO outcomes in AAA.

### Frameworks of flourishing

Flourishing in positive psychology highlights health as more than just the absence of disease.^16^ It includes positive emotions, purpose, resilience, and social connections.^17^ Instead of focusing only on deficits, flourishing reflects overall well-being, even in the face of chronic illness.^17^ All current models of flourishing agree that flourishing entails both *hedonic* (positive feeling) and *eudaimonic* (positive functioning) aspects of well-being.

Current dominant models that define flourishing include those by Keyes, Huppert, Diener, and Seligman. Keyes’ model^18^ conceptualizes flourishing as the absence of mental illness combined with high emotional, psychological, and social well-being. States of flourishing refer to the absence of psychopathology and high well-being ratings across various domains.^18^ Huppert’s model^17^ links high well-being with positive mental health, in contrast to common disorders like depression and anxiety. It includes 10 flourishing elements: competence, emotional stability, engagement, meaning, optimism, positive emotion, relationships, resilience, self-esteem, and vitality. Diener’s model^19^ links flourishing to success in relationships, self-esteem, purpose, and optimism.^19^ An eight-item scale measures these, combined with the Scale of Positive and Negative Experience to generate a composite score.^20^ This assesses the proportion of negative versus positive emotions over the past 4 weeks. Seligman’s model^21^ led to PERMA, representing positive emotion, engagement, relationships, meaning, and accomplishment,^21^ and was inspired by Aristotle, who believed flourishing involved developing ‘our truly human capacities.’

There is no one gold standard to measure flourishing, but commonly used tools to measure it include the Mental Health Continuum-Short Form, the Flourishing Scale,^18^ and the PERMA-Profiler.^22^

## Methods

### Search strategy

The literature search involved a Medline search (November 22, 2024) to identify works related to PROs in AAA populations. The search strategy and MeSH terms are summarized in Supplemental Table S1. The search was updated on February 1, 2025. Two reviewers (KS and CM) independently screened abstracts and titles, resolving any conflicts with a third reviewer (CT). Full-text papers were then obtained and screened for further study selection. Citation chasing was performed with full-text papers. Covidence^23^ software was used for screening and selection of articles.

Key papers were hand-selected by the research team from original studies and reviews that reported on conceptual frameworks of flourishing in patient and integrated care settings. Search terms included ‘conceptual,’ ‘model,’ and ‘flourishing.’

### Eligibility criteria and study selection

For the PRO literature, the following inclusion and exclusion criteria were adopted based on prior work.^24^ Studies were included if they: (1) collected quantitative or qualitative data on disease-specific PROs in AAA; (2) collected generic health status quantitative data in patients with AAA; (3) used questions that were developed de novo (i.e., not built/adapted from questions on existing generic or disease-specific patient-reported outcome measures [PROMs]); (4) collected information on treatment goals and preferences; (5) collected quantitative or qualitative data on patient-centered outcomes; (6) collected quantitative or qualitative data on disease impact; (7) collected information about psychological well-being in patients with AAA; and (8) were conducted between January 1, 1980 and February 1, 2025. Studies were excluded if they were an editorial, commentary, or letter, or if the study was not in English. For the flourishing papers, no a priori inclusion or exclusion criteria were applied, except for the requirement of English language. As our narrative literature review did not involve human subjects or de novo data collection, no institutional review board approval was required.

### Data extraction

Data from the included studies were extracted into prestructured tables in Microsoft Word, including authorship, publication year, study design, population, main outcome, instruments, and key findings. Data on the psychometric properties of the PRO instruments were collected, including reliability, validity, ability to detect change, and interpretability, based on US Department of Health and Human Services Food and Drug Administration (FDA) guidance.^25^ Flourishing elements from previously established frameworks^17,18,20,21^ were identified during data extraction.

The instruments were divided into disease-specific health questionnaires, generic health questionnaires, and psychological screening questionnaires, based on validated populations and their intended purpose.

### Review of psychometric properties of patient-reported outcomes (PROs) in individuals with abdominal aortic aneurysm (AAA)

The field of PRO assessment in medical populations primarily adopts the perspective of the Classical Test Theory, which provides a well-defined and structured work process for designing reliable instruments that have adequate validity. For this review, we followed the industry guidance for PROs developed by the FDA,^25^ which also adopts Classical Test Theory for quality evaluation. Although it is beyond the scope of this review to do an extensive psychometric evaluation, we do comment on aspects of reliability and validity, their sensitivity to change, and whether meaningful frameworks for clinically important differences have been developed in the target population for the instrument’s intended use. Finally, we also comment on availability and user-friendliness, given item length, and access to the questionnaires.

## Results

### Search results and data extraction of study characteristics

**Figure 1.**
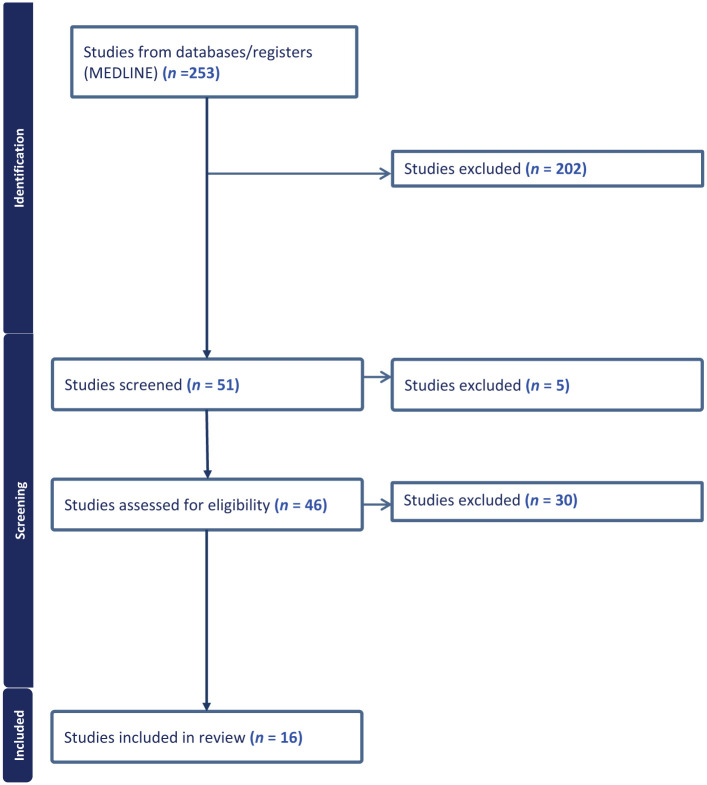
Flow diagram for literature search to identify studies on assessing quality of life and flourishing in the different clinical phenotypes of abdominal aortic aneurysm. Details of the included study characteristics and reasons for exclusion are provided in Supplemental Tables S2 and S3, respectively.

The database search resulted in 253 articles, all screened for title and abstract. After a second review by a third reviewer, 47 studies underwent full-text review, and 16 studies met the inclusion criteria (Figure 1). Details of the included study characteristics and reasons for exclusion are provided in Tables S2 and S3, respectively.

Most of the included studies were conducted in the United Kingdom (*n* = 7), followed by the United States (*n* = 3) and The Netherlands (*n* = 3), Denmark (*n* = 1), Italy (*n* = 1), and Sweden (*n* = 1). Sample sizes ranged from 50 to 9030 with a total of 18,216; one of the studies^26^ did not provide a sample size. The male population was predominantly represented in this review, comprising approximately 64% (11,776 out of 18,357) of the total studied individuals with AAA (Table 1).

**Table 1.** Disease-specific patient-reported outcome measures (PROMs) for abdominal aortic aneurysm by clinical phenotype and psychometric properties.

Instruments	AAA clinical phenotype		
	Screening	Surveillance	Elective surgery
**Disease-specific health questionnaires**			
**AneurysmDQoL**^40^ (22 domain-specific items)		×	×
**AneurysmSRQ**^40^ (24 items, 6 domains)		×	×
**AneurysmTSQ**^40^ (11 items,1 domain)		×	×
**COS-AAA**^27^ (Part I: 70 items, 18 domains; Part II: 21 items, 5 domains)	×	×	
**Aortic Aneurysm QoL**^40^ (54 items, 2 domains)		×	×
**Generic health questionnaires**			
**EQ-5D-5L**^53^ (5 items, 5 domains)	×	×	×
**5Q-VAS**^54^ (5 item, 5 domains)	×	×	×
**SF-36**^55^ (36 items, 8 domains)			×
**PCQ**^26^ (24 items, 6 domains)	×		
**PACES-S**^56^ (18 items, 4 domains)			×
**WIQ**^34^ (24 items, 3 domains)			×
**IIEF**^34^ (15 items, 5 domains)			×
**WHOQOL-BREF**^30^ (26 items, 4 domains)			×
**Psychological screening**			
**HADS**^36^ (14 items, 2 domains : **HADS-A** [7 items]; **HADS-D** [7 items)]		×	
**CES-D 16**^30^ (16 items, 4 domains)			×

AAA, abdominal aortic aneurysm; AneurysmDQoL, Aneurysm-Dependent Quality of Life; AneurysmSRQ, Aneurysm-Symptom Rating Questionnaire; AneurysmTSQ, Aneurysm-Treatment Satisfaction Questionnaire; CES-D 16, Center for Epidemiologic Studies Depression 16 questionnaire; COS-AAA, Consequences of Screening in Abdominal Aortic Aneurysm questionnaire; EQ-5D-5L, EuroQoL 5-Dimension 5-level questionnaire; EQ-VAS, EuroQoL Visual Analogue Scale; HADS-A, Hospital Anxiety and Depression Scale (HADS), anxiety domain; HADS-D, Hospital Anxiety and Depression Scale (HADS), depression domain; IIEF, International Index of Erectile Function; PACES-S, Physical Activity Enjoyment Scale; PCQ, Psychological Consequences Questionnaire; QoL, quality of life; SF-36, 36-Item Short Form health survey; WHOQOL-BREF, World Health Organization Quality of Life-Brief; WIQ, Walking Impairment Questionnaire.

**Table 2.** Disease-specific questionnaires by dimensions and flourishing elements from models.

Scales	Dimensions	Flourishing elements										
		Psychopathology	Emotional stability	Competence	Engagement	Meaning	Optimism	Positive emotion	Positive Relationships	Resilience	Self esteem	Vitality
**AneurysmDQoL**^40^ (22 domain-specific items)	Leisure				×			×				
	Work			×		×						
	Long distance journeys											
	Holidays											
	Do physically											
	Family life								×			
	Friendships and social life								×			
	Closes personal relationships								×			
	Sex life											
	Getting out and about											
	Household tasks											
	Do things for others											
	Enjoy food							×				
	Feelings about the future	×				×	×					
	Finance											
	Having to depend on others											
	Health											
	Others fuss or worry	×										
	Energy											×
	Physical discomfort											
	Anxiety	×										
	Think clearly, concentrate and remember											
**AneurysmSRQ**^40^ (24 items, 6 domains)	Emotion		×					×				
	Appetite											
	Lower limb											
	Cognitive											
	General malaise											×
	Gastrointestinal symptoms											
**AneurysmTSQ**^40^ (11 items, 1 domain)	Treatment satisfaction (before and after)											
**COS-AAA**^27^ (Part I: 70 items and 18 domains; Part II^a^: 21 items and 5 domains)	Anxiety	×										
	Behavioral	×										
	Dejection	×										
	Sleep	×										×
	Uncertainty about the results of the ultrasound examination	×										
	Change in body perception										×	
	Guilt	×										
	Fear and powerlessness	×										
	Negative experiences from the examination	×										
	Emotional reactions	×										
	Change in lifestyle											
	Better not knowing											
	Fear of rupture	×										
	Sexuality								×			×
	Information											
	Stigmatized											
	Self-blamed											
	Regretful											
	Existential values^a^											
	Relaxed / calm^a^											
	Social relations^a^											
	Impulsivity^a^											
	Empathy^a^											
**Aortic Aneurysm QoL**^41^ (54 items, 2 domains)	Emotional impact	×	×					×	×			×
	Behavior change											

AAA, abdominal aortic aneurysm; AneurysmDQoL, Aneurysm-Dependent Quality of Life; AneurysmSRQ, Aneurysm-Symptom Rating Questionnaire; AneurysmTSQ, Aneurysm-Treatment Satisfaction Questionnaire; COS-AAA, Consequences of Screening in Abdominal Aortic Aneurysm questionnaire; QoL, quality of life.

**Figure 2.**
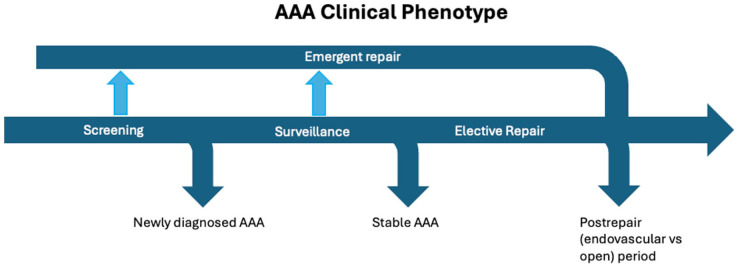
Phenotypic evolution and intervention pathways in the clinical course of abdominal aortic aneurysm (AAA).

Ten studies were prospective cohorts^26–35^ and six were retrospective cohorts.^36–41^ Twelve studies included elective repair AAA clinical phenotype patients (open or endovascular intervention), four studies included screening AAA populations, and three studies included a surveillance AAA population. No study included individuals undergoing emergency repair for AAA (Figure 2).

### Disease-specific health questionnaires

The five identified disease-specific health questionnaires in this review were: the Aneurysm-Dependent Quality of Life Questionnaire (AneurysmDQoL), the Aneurysm Symptom Rating Questionnaire (AneurysmSRQ), the Aneurysm Treatment Satisfaction Questionnaire (AneurysmTSQ), the Consequences of Screening in Abdominal Aortic Aneurysm questionnaire (COS-AAA), and the Aortic Aneurysm Quality of Life (QoL) instrument.^27,40,41^ The overview of PRO domains, psychometric properties, and expansion of evidence are detailed in Table S5.

The AneurysmDQoL, AneurysmSRQ, and AneurysmTSQ are three new aneurysm-specific questionnaires created by Peach et al., with the goal to provide robust, separate assessments of quality of life, symptoms, and treatment satisfaction for use in clinical practice, audit, and research.^40,42^ The three questionnaires were applied simultaneously to the same patient cohort. For validity, patients were recruited from four hospitals and had undergone AAA repair (open or endovascular) within 24 months, or were enrolled in preoperative surveillance.^42^ Focus groups on patients’ experiences on quality of life, symptoms, and treatment satisfaction at each treatment stage were conducted using open-ended questions. The questions were chosen from an existing item bank and updated after group sessions, then checked by a linguistic specialist. The inclusion of items was based on how often topics were discussed in groups. Peach et al. evaluated the psychometric properties of these three new condition-specific questionnaires, and a detailed psychometric validation was described before.^40^ They sent these questionnaires to 297 patients with AAA, or to those who had undergone AAA repair, with a 69% response rate.^40^ Information about the evaluation of the psychometric properties of these three instruments is detailed in Tables S4 and S5. These instruments can be licensed through a formal agreement with Health Psychology Research Ltd.^43^

In the AneurysmDQoL questionnaire, focus groups were used to identify 22 potentially impacted quality of life domains, assessed with Likert scales for opinions and a free-text option for other affected areas.^40^ The AneurysmSRQ contains 24 items addressing commonly reported symptoms across 6 domains (emotion, appetite, lower limb, cognitive, general malaise, and gastrointestinal symptoms).^40^ Focus groups identified symptoms related to the aneurysm or its repair: pain (leg, lower back, abdomen, buttock), swelling, numbness, weakness, heaviness in the lower limb, low mood, and weight loss.^40^ The AneurysmTSQ^40^ focused on elements of overall treatment satisfaction, and it was developed based on the Diabetes Treatment Satisfaction Questionnaire^44^ and other associated questionnaires.^45,46^ It contains 11 items rated on a seven-point scale, with higher scores indicating greater satisfaction with treatment, convenience, discomfort, information, feedback, support, follow-up, and monitoring of treatment demands.^42^

Brodersen et al.^27^ developed the Consequences of Screening in Abdominal Aortic Aneurysm (COS-AAA) questionnaire, an adaptation of previously validated screening consequence questionnaires used in breast cancer, lung cancer, and cervical cancer.^47,48,49^ Only men undergoing AAA screening participated in its development through small group interviews based on existing COS items and their lived experiences. This instrument followed a partial credit Rasch model^50^ to assess item performance, with scale homogeneity and differential item functioning evaluated using Andersen’s conditional likelihood ratio test.^51^ The detailed psychometric validation of this questionnaire is described elsewhere.^27^ A draft version was mailed to men, and 158 (63%) men with screening-detected AAA and 275 (55%) men with normal screening results returned the questionnaire.^27^ Finally, COS-AAA ended up consisting of two parts: Part I includes 18 domains with more than 70 items and Part II includes five domains with 21 items.^27^ The questionnaire is available online in the study’s supplemental material; however, initial validation work was performed in Swedish and Danish languages.^27^

**Figure 3.**
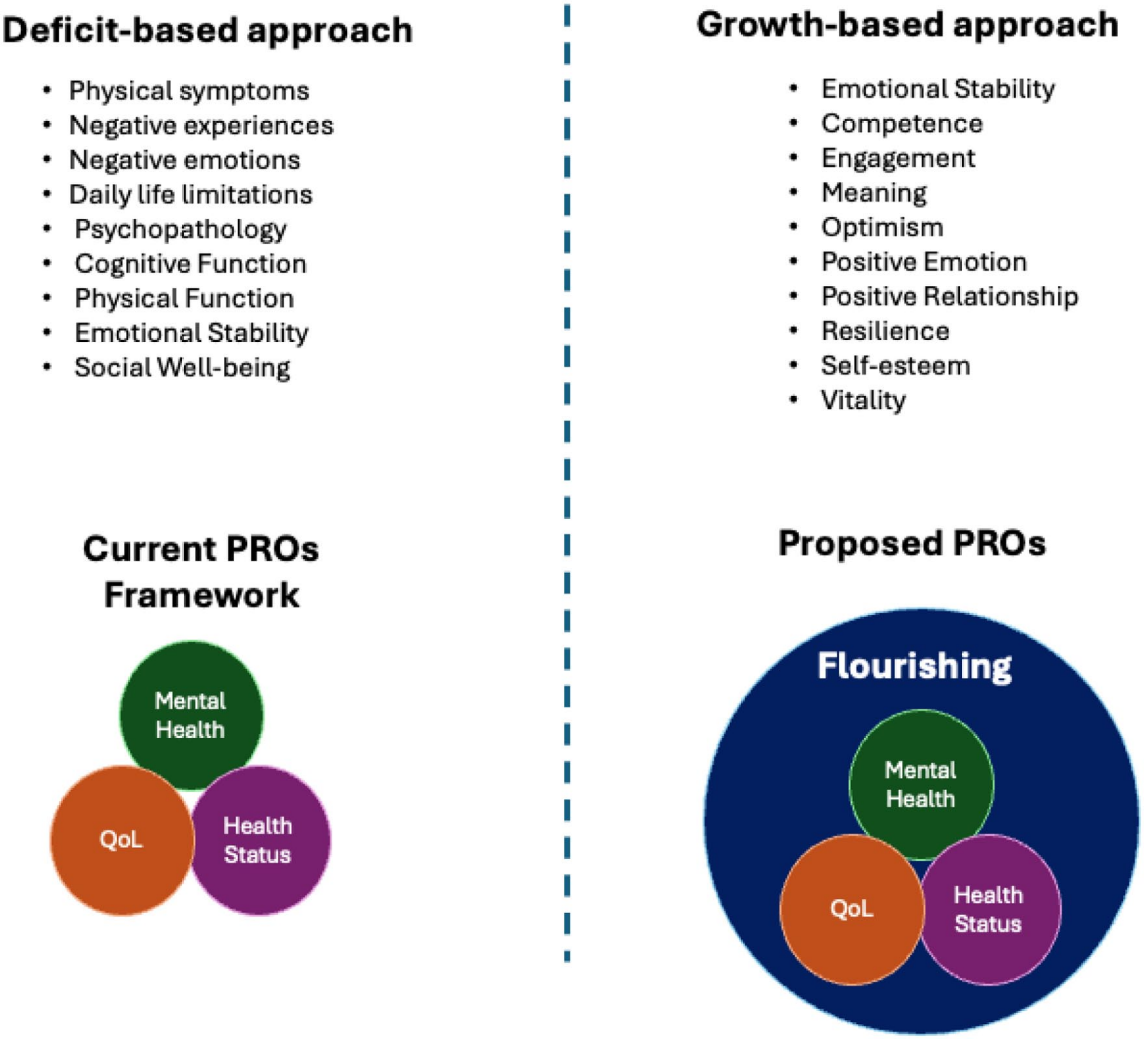
Shifting from a deficit-based to a growth-oriented framework in patient-reported outcomes assessment. PROs, patient-reported outcomes; QoL, quality of life.

The disease-specific Aortic Aneurysm QoL survey was developed by Suckow et al. in US patients who had undergone AAA surgical repair (open or endovascular).^41^ This instrument was developed between 2011 and 2013 and validated between 2013 and 2014. It measures two AAA-specific domains of quality of life: emotional impact and behavioral change. Two versions were created: one for patients who had undergone AAA repair surgery (55 questions) and one for patients living with AAA (62 questions). Surveys were mailed to patients along with a cash incentive and the 12-Item Short Form health Survey (SF-12) generic health status survey. The surveys were written in simple, nontechnical language to ensure clarity and reliable responses, prioritizing cost-effectiveness and ease of completion.^41^ The detailed psychometric validation of this questionnaire is reported elsewhere, including the use of three patient focus groups and two vascular surgeon groups to establish content validity.^41,50^ The questionnaire is freely accessible online^41^ (Figure 3).

### Generic health questionnaires

Several generic health questionnaires that have been used in individuals with AAA were identified: the EuroQoL-5D, the 36-Item Short Form health survey (SF-36), the Physical Activity Enjoyment Scale (PACES-S), and the World Health Organization Quality of Life-Brief (WHOQOL-BREF) instrument.^30,52-56^ The overview of PROM domains, psychometric properties, and expansion of evidence are detailed in Table S5.

Other unidimensional instruments not specifically designed for AAA but used in patients undergoing screening and/or surgical procedure repair include the Psychological Consequences Questionnaire (PCQ),^26^ which evaluates the psychosocial impact of mammography screening on a patient, but the questionnaire items were modified for AAA screening. A unidimensional instrument was the Walking Impairment Questionnaire (WIQ),^34^ which evaluates walking ability, especially in patients with peripheral artery disease (PAD).^57^ This instrument has three domains and 24 items, scored 0–4. The final score is a percentage from 0% to 100%.^57^ This questionnaire was given to a postsurgical AAA group. The International Index of Erectile Function (IIEF)^34^ evaluates male sexual dysfunction in a comprehensive manner. This instrument contains 15 items assessing five domains, such as erectile dysfunction, orgasmic function, sexual desire, intercourse satisfaction, and overall satisfaction.^58^ This questionnaire was also administered to male patients who underwent AAA repair.^58^

### Psychopathology questionnaires

The reviewed studies mainly targeted anxiety and depression, using either specific subscales for *Diagnostic and Statistical Manual of Mental Disorders* criteria or symptom assessments within broader questionnaires. Some disease-specific health questionnaires^27,40,41^ evaluated symptoms of anxiety and depression in a general manner. The Aortic Aneurysm QoL^41^ survey explores anxiety and depression symptoms via two questions for patients with AAA: (1) How often do you feel anxious now compared to before your AAA repair/diagnosis? and (2) How often do you feel sad now compared to before your AAA repair/diagnosis? Finally, the COS-AAA also evaluated anxiety symptoms over 2 weeks, including worry, worry about the future, and being scared, irritable, nervous, terrified, shocked, etc.^27^

Two psychopathology screening instruments used in AAA populations were identified: the Hospital Anxiety and Depression Scale (HADS) and the Center for Epidemiologic Studies Depression 16 questionnaire (CES-D 16).^30,36^

The HADS, which assesses depression symptoms (HADS-D) and anxiety symptoms (HADS-A), is a screening tool for psychological distress that may require further evaluation and treatment in medical populations.^36^ This questionnaire has 14 items in two subscales—Anxiety and Depression—each with seven items, totaling 42 points (21 per subscale). Higher scores indicate greater anxiety or depression.^59^ Using this questionnaire in 752 patients undergoing AAA surveillance, clinically relevant anxiety levels were revealed in 14.6% of women and 5.5% of men.^36^

The CES-D 16^30^ is a short version of the original CES-D, designed for epidemiological research and public health studies, and evaluates depressive symptoms in general and clinical populations. It is intended as a screening not a diagnosis tool. The 16-item questionnaire covers four domains: depressed affect, positive affect, somatic symptoms, and interpersonal problems. It was used postsurgery on an AAA population and evaluated alongside the presence of delirium, and the authors found that patients who developed delirium had significantly higher depressive symptoms scores after 12 months compared to patients who did not develop delirium (26 vs 19; *p* = 0.027).^30^

### Flourishing in AAA-specific health questionnaires

Five disease-specific health questionnaires were identified that evaluate flourishing elements in different AAA phenotypes (AneurysmDQoL, AneurysmSRQ, AneurysmTSQ, COS-AAA, and the Aortic Aneurysm QoL instrument).

The questionnaires AneurysmDQoL, AneurysmSRQ, and AneurysmTSQ assessed the impact of AAA on physical, psychological, and social well-being. Although they do not directly measure flourishing, some address aspects of well-being that contribute to flourishing in patients with AAA and during postsurgery treatment. AneurysmDQoL assessed AAA’s impact on emotional well-being, independence, daily activities, and patients’ ability to maintain a fulfilling life despite their clinical phenotype. The AneurysmSRQ showed no signs of flourishing; it focuses on AAA symptom severity, frequency, and physical burden mostly, and the AneurysmTSQ measures patient satisfaction with treatment, including expectations, perceived benefits, and concerns about future health.^40^

In COS-AAA, elements of flourishing such as psychological well-being, social engagement, and satisfaction were present. It also measures anxiety, depression, and emotional distress to assess daily functioning and perceptions of well-being in patients with AAA.^27^

The Aortic Aneurysm QoL^41^ survey reflects flourishing by evaluating well-being, psychological health, and social engagement. It measures how patients handle daily tasks, emotional distress, social ties, and their health perceptions, aiming to increase personal growth, life satisfaction, and health control.^41^

## Discussion

This review included 16 studies on PRO instruments measuring health, quality of life, mental health, and flourishing in patients with AAA. Five disease-specific tools were identified: some focus on surveillance and postelective repair (AneursymDQoL, AneurysmSRQ, AneurysmTSQ, Aortic Aneurysm QoL) and one on screening populations (COS-AAA). Most PROs emphasized physical function, daily activities, independence, symptoms across various domains (emotional, appetite, cognitive, gastrointestinal, sleep, sexual), lifestyle changes, and social relationships. Limited accessibility of disease-specific questionnaires is notable, with only two of five freely available, and item response burden ranging from 11 to 70 items. Standardized questionnaires for psychopathology focused mainly on symptoms of anxiety and depression. Additionally, articles showed limited, indirect assessment of elements of flourishing in patients with AAA, and the construct was not considered during questionnaire development. Validation efforts using Classical Test Theory were in the early stages for most questionnaires. However, five had undergone more advanced validation, particularly in individuals with AAA undergoing screening and/or surgical repair.^27,40,41^ Nonetheless, further research is needed to validate these questionnaires in larger and more diverse AAA populations and clinical phenotypes to confirm their broader applicability and effectiveness in assessing health outcomes across different AAA populations.

As more people face chronic cardiovascular disease, including those affected by AAA, an opportunity presents itself to reassess PRO evaluation methods reflective of diverse aspects of patient-centered functioning. The field has mainly used traditional instruments to measure disease impact but has not considered broader aspects of thriving across ages, especially where populations face disability and chronic disease, and psychosomatic distress even in the asymptomatic phases of the disease.^60^ Focusing on deficit approaches to evaluate functioning may not provide a full picture of people’s true functioning, including their ability to flourish, which encompasses the ability to experience positive emotions, seek purpose, experience optimism, feel competent, and maintain emotional stability.^61–63^ In addition, other dynamic needs of PRO assessments were identified, including the ability to account for how other comorbidities impact functioning and evolving needs as people move through the phases of their life while managing chronic disease. For AAA, individuals may spend years in the surveillance phase^64,65^ and are able to lead thriving lives, but for others, their enjoyment and sense of purpose may be impacted by the knowledge of having AAA and the thought of possible rupture.

Current instruments may only detect psychopathology and physical deficits in individuals with AAA and may not distinguish between thriving and nonthriving individuals. Clinical phenotypes for AAA are dynamic, so assessment items should reflect these changes. Static instruments based on Classical Test Theory may not fully capture all states and needs without overburdening patients with long questionnaires,^66^ limiting their routine clinical use. Newer frameworks have been developed that use adaptive testing strategies based on item response theory frameworks^67^ and can incorporate previously measured traits (e.g., clinical staging, other comorbidities) that may impact individuals’ responding tendencies.^68^ Such approaches incorporate trait-level patient features and tailor questions based on responses, creating a more efficient, personalized method for PRO assessment that targets relevant aspects for each individual at their specific age and clinical phenotype stage. Successful adaptive testing examples exist within the Patient-Reported Outcomes Measurement Information System (PROMIS) scales, which are capable of making adaptations for the various medical conditions individuals are dealing with,^69–72^ and a similar approach has been adopted for individuals with diabetes.^73^

As chronic diseases affect more people, defining their functioning solely by deficits is not sustainable. Focusing only on limitations overlooks human potential, adaptation, growth, and resilience despite the physical challenges caused by disease. Growth-based approaches are not new. In the space of cancer research, posttraumatic growth is a well-studied outcome in those living with a cancer diagnosis.^74,75^ Cardiovascular disease management has yet to incorporate posttraumatic growth and positive psychology. Doing so could enhance life satisfaction, adherence to lifestyle changes, and clinical outcomes, and would fit better in an integrated, whole-person, chronic disease management context. It also enables evidence-based interventions to promote well-being in individuals with chronic illnesses. Including these aspects in PROs could quantify benefits and thriving, beyond just measuring deficits or harm (e.g., in the scenario of screening).

**Figure 4.**
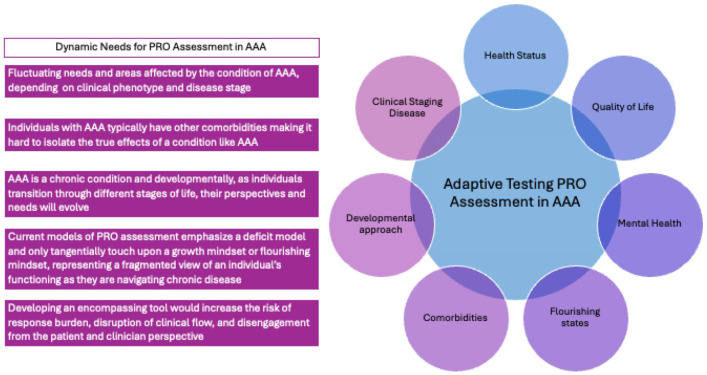
Dynamic considerations for patient-reported outcomes assessment in abdominal aortic aneurysm. AAA, abdominal aortic aneurysm; PRO, patient-reported outcomes.

Building on the gaps identified in current PRO instruments, future AAA-specific assessment approaches may benefit from integrating contemporary measurement methodologies (item response theory-based models and computerized adaptive testing) alongside broader constructs of flourishing and positive psychological well-being, as well as the expansion of items to capture currently underrepresented clinical phenotypes such as the patient with AAA undergoing emergency repair. Our proposed framework calls for future steps, including developing items with growth-based frameworks and more diverse and complex clinical scenarios, and building a test bank for large-scale trials. This will gather trait-based data, enabling precise adaptive testing (Figure 4). The framework presented in this work can be transposed to other cardiovascular chronic conditions and by using the AAA condition as a user-case scenario, we hope to lay out a thought framework that may inspire evolving approaches of PRO assessment across cardiovascular conditions to integrate the construct of flourishing and for PROs to be designed as adaptive tools that reflect the highly individualized needs and stages of the disease process, against an individual’s overall functioning. Rather than replacing existing tools, this direction aligns with successful applications in other clinical fields. NIH PROMIS adaptive instruments are widely used across oncology, rheumatology, and geriatrics to capture domains such as emotional support, meaning and purpose, positive affect, fatigue, and physical function with high precision and reduced patient burden. Similarly, flourishing-oriented constructs have been incorporated into survivorship research in oncology and into cardiovascular behavioral science, where characteristics such as optimism, emotional stability, and purpose have been associated with improved recovery trajectories, adherence to treatment, and long-term outcomes.

### Limitations

The limitations in this review are the following. First, the disease-specific questionnaires may not capture broader mental-health issues or comorbidities which are common in patients with AAA. Second, validation of disease-specific questionnaires across diverse patient populations and stages of disease, including postsurgical emergency interventions, is essential to ensure their reliability and applicability in various clinical contexts. Third, the review did not include health economic expressions of potential harm related to AAA screening, which are expressed as utilities derived from generic health status instruments such as the EQ-5D. Finally, most of the studies (11/16) included a predominantly male patient population (64%), highlighting a clear gap in understanding the specific experiences and challenges faced by women living with this condition, largely due to the small number of female participants in existing studies.

## Conclusion

This review shows that only a few AAA-specific PRO tools are in the early stages of development using the Classical Test Theory framework, and a one-size-fits-all approach may not meet the needs of diverse clinical phenotypes. Flourishing experiences—such as positive emotions and relationships, emotional stability, self-esteem, optimism, engagement, resilience, competence, and meaning—are not currently explored in AAA populations, despite their relevance for chronic disease care. As we move toward more integrated, whole-person approaches of care for aging populations with multimorbidity, a broader conceptual framework for PROs is needed, one that goes beyond the absence of disability to assess whether individuals can enjoy and find meaning in life despite chronic disease. This shift requires refining existing tools, incorporating behavioral assessments into clinical pathways, and transitioning to adaptive, item response theory-based approaches aligned with precision medicine.

## Supplemental Material

sj-docx-1-vmj-10.1177_1358863X261417234 – Supplemental material for Evolving dynamic needs for patient-reported outcomes assessment in individuals with an abdominal aortic aneurysm (AAA): A systematic review Supplemental material, sj-docx-1-vmj-10.1177_1358863X261417234 for Evolving dynamic needs for patient-reported outcomes assessment in individuals with an abdominal aortic aneurysm (AAA): A systematic review by Kim G Smolderen, Christiany Tapia, Bernard Dennis, Santiago Callegari, Marie Dahl, Jes S Lindholt, Isabelle Van Herzeele, Gaëlle Romain and Carlos Mena-Hurtado in Vascular Medicine
