# Supplementary material for: Evolving dynamic needs for patient-reported outcomes assessment in individuals with an abdominal aortic aneurysm (AAA): A systematic review
Source: Vasc Med. 2026 Mar 19;31(2):232–45. doi: 10.1177/1358863X261417234 (PMC13109597; doi:10.1177/1358863X261417234)
Supplement: sj-docx-1-vmj-10.1177_1358863X261417234 – Supplemental material for Evolving dynamic needs for patient-reported outcomes assessment in individuals with an abdominal aortic aneurysm (AAA): A systematic review [file sj-docx-1-vmj-10.1177_1358863X261417234.docx]

**SUPPLEMENTAL MATERIAL**

**Supplemental Table S1.** Search Terms

**Supplemental Table S2.** Table of Articles Included in the Review

**Supplemental Table S3.** Table of Excluded Studies with Reasons for Exclusion

**Supplemental Table S4.** Evaluation of Psychometric Properties of Patient-Reported Outcome (PRO) Instruments in Clinical Trials based on the U.S. Department of Health Guidance

**Supplemental Table S5.** Overview of patient-reported outcome measure (PROM) domains, psychometric properties, and expansion of the evidence base

**Table S1.** Literature Search details in Medline.

|  | **#** | **Search Terms** |
| --- | --- | --- |
| **Medline** | | |
|  | 1 | ("aortic aneurysm, abdominal"[MeSH Terms] OR ("aortic"[All Fields] AND "aneurysm"[All Fields] AND "abdominal"[All Fields]) OR "abdominal aortic aneurysm"[All Fields] OR ("abdominal"[All Fields] AND "aortic"[All Fields] AND "aneurysm"[All Fields]) OR (("aortic aneurysm, abdominal"[MeSH Terms] OR ("aortic"[All Fields] AND "aneurysm"[All Fields] AND "abdominal"[All Fields]) OR "abdominal aortic aneurysm"[All Fields] OR ("abdominal"[All Fields] AND "aneurysm"[All Fields]) OR "abdominal aneurysm"[All Fields]) AND ("repairability"[All Fields] OR "repairable"[All Fields] OR "repaire"[All Fields] OR "repaired"[All Fields] OR "repairment"[All Fields] OR "wound healing"[MeSH Terms] OR ("wound"[All Fields] AND "healing"[All Fields]) OR "wound healing"[All Fields] OR "repair"[All Fields] OR "repairing"[All Fields] OR "repairs"[All Fields])) OR (("aorta"[MeSH Terms] OR "aorta"[All Fields] OR "aortic"[All Fields] OR "aortics"[All Fields]) AND ("dilatable"[All Fields] OR "dilatated"[All Fields] OR "dilatating"[All Fields] OR "dilatation"[MeSH Terms] OR "dilatation"[All Fields] OR "dilatations"[All Fields] OR "dilate"[All Fields] OR "dilation"[All Fields] OR "dilations"[All Fields] OR "dilatative"[All Fields] OR "dilatator"[All Fields] OR "dilatators"[All Fields] OR "dilated"[All Fields] OR "dilates"[All Fields] OR "dilating"[All Fields] OR "dilator"[All Fields] OR "dilators"[All Fields])) OR (("aorta"[MeSH Terms] OR "aorta"[All Fields] OR "aortic"[All Fields] OR "aortics"[All Fields]) AND ("abdomen"[MeSH Terms] OR "abdomen"[All Fields] OR "abdominal"[All Fields] OR "abdominally"[All Fields] OR "abdominals"[All Fields]) AND ("ruptur"[All Fields] OR "rupture"[MeSH Terms] OR "rupture"[All Fields] OR "ruptured"[All Fields] OR "ruptures"[All Fields] OR "rupturing"[All Fields]))) AND "2014/05/20 00:00":"3000/01/01 05:00"[Date - Publication] |
|  | 2 | "questionnair"[All Fields] OR "questionnaire s"[All Fields] OR "surveys and questionnaires"[MeSH Terms] OR ("surveys"[All Fields] AND "questionnaires"[All Fields]) OR "surveys and questionnaires"[All Fields] OR "questionnaire"[All Fields] OR "questionnaires"[All Fields] OR ("instrument"[All Fields] OR "instrument s"[All Fields] OR "instrumentation"[MeSH Subheading] OR "instrumentation"[All Fields] OR "instruments"[All Fields] OR "instrumented"[All Fields] OR "instrumenting"[All Fields]) OR "tool"[All Fields] |
|  | 3 | "quality of life"[MeSH Terms] OR ("quality"[All Fields] AND "life"[All Fields]) OR "quality of life"[All Fields] OR ("patient reported outcome measures"[MeSH Terms] OR ("patient"[All Fields] AND "reported"[All Fields] AND "outcome"[All Fields] AND "measures"[All Fields]) OR "patient reported outcome measures"[All Fields] OR ("patient"[All Fields] AND "reported"[All Fields] AND "outcomes"[All Fields]) OR "patient reported outcomes"[All Fields]) OR ("health status"[MeSH Terms] OR ("health"[All Fields] AND "status"[All Fields]) OR "health status"[All Fields]) OR ("patient-centered"[All Fields] AND ("outcome"[All Fields] OR "outcomes"[All Fields])) OR ("quality of life"[MeSH Terms] OR ("quality"[All Fields] AND "life"[All Fields]) OR "quality of life"[All Fields] OR ("health"[All Fields] AND "related"[All Fields] AND "quality"[All Fields] AND "life"[All Fields]) OR "health related quality of life"[All Fields]) |
|  | 4 | ("aortic aneurysm, abdominal"[MeSH Terms] OR ("aortic"[All Fields] AND "aneurysm"[All Fields] AND "abdominal"[All Fields]) OR "abdominal aortic aneurysm"[All Fields] OR ("abdominal"[All Fields] AND "aortic"[All Fields] AND "aneurysm"[All Fields]) OR (("aortic aneurysm, abdominal"[MeSH Terms] OR ("aortic"[All Fields] AND "aneurysm"[All Fields] AND "abdominal"[All Fields]) OR "abdominal aortic aneurysm"[All Fields] OR ("abdominal"[All Fields] AND "aneurysm"[All Fields]) OR "abdominal aneurysm"[All Fields]) AND ("repairability"[All Fields] OR "repairable"[All Fields] OR "repaire"[All Fields] OR "repaired"[All Fields] OR "repairment"[All Fields] OR "wound healing"[MeSH Terms] OR ("wound"[All Fields] AND "healing"[All Fields]) OR "wound healing"[All Fields] OR "repair"[All Fields] OR "repairing"[All Fields] OR "repairs"[All Fields])) OR (("aorta"[MeSH Terms] OR "aorta"[All Fields] OR "aortic"[All Fields] OR "aortics"[All Fields]) AND ("dilatable"[All Fields] OR "dilatated"[All Fields] OR "dilatating"[All Fields] OR "dilatation"[MeSH Terms] OR "dilatation"[All Fields] OR "dilatations"[All Fields] OR "dilate"[All Fields] OR "dilation"[All Fields] OR "dilations"[All Fields] OR "dilatative"[All Fields] OR "dilatator"[All Fields] OR "dilatators"[All Fields] OR "dilated"[All Fields] OR "dilates"[All Fields] OR "dilating"[All Fields] OR "dilator"[All Fields] OR "dilators"[All Fields])) OR (("aorta"[MeSH Terms] OR "aorta"[All Fields] OR "aortic"[All Fields] OR "aortics"[All Fields]) AND ("abdomen"[MeSH Terms] OR "abdomen"[All Fields] OR "abdominal"[All Fields] OR "abdominally"[All Fields] OR "abdominals"[All Fields]) AND ("ruptur"[All Fields] OR "rupture"[MeSH Terms] OR "rupture"[All Fields] OR "ruptured"[All Fields] OR "ruptures"[All Fields] OR "rupturing"[All Fields]))) AND "2014/05/20 00:00":"3000/01/01 05:00"[Date - Publication] AND ("questionnair"[All Fields] OR "questionnaire s"[All Fields] OR "surveys and questionnaires"[MeSH Terms] OR ("surveys"[All Fields] AND "questionnaires"[All Fields]) OR "surveys and questionnaires"[All Fields] OR "questionnaire"[All Fields] OR "questionnaires"[All Fields] OR ("instrument"[All Fields] OR "instrument s"[All Fields] OR "instrumentation"[MeSH Subheading] OR "instrumentation"[All Fields] OR "instruments"[All Fields] OR "instrumented"[All Fields] OR "instrumenting"[All Fields]) OR "tool"[All Fields]) AND ("quality of life"[MeSH Terms] OR ("quality"[All Fields] AND "life"[All Fields]) OR "quality of life"[All Fields] OR ("patient reported outcome measures"[MeSH Terms] OR ("patient"[All Fields] AND "reported"[All Fields] AND "outcome"[All Fields] AND "measures"[All Fields]) OR "patient reported outcome measures"[All Fields] OR ("patient"[All Fields] AND "reported"[All Fields] AND "outcomes"[All Fields]) OR "patient reported outcomes"[All Fields]) OR ("health status"[MeSH Terms] OR ("health"[All Fields] AND "status"[All Fields]) OR "health status"[All Fields]) OR ("patient-centered"[All Fields] AND ("outcome"[All Fields] OR "outcomes"[All Fields])) OR ("quality of life"[MeSH Terms] OR ("quality"[All Fields] AND "life"[All Fields]) OR "quality of life"[All Fields] OR ("health"[All Fields] AND "related"[All Fields] AND "quality"[All Fields] AND "life"[All Fields]) OR "health related quality of life"[All Fields])) |

**Table S2.** Matrix of Articles Included in the Review

| **Author, Year, Location** | **Type of study** | **AAA Clinical Phenotype** | **Population *n* (%)** | **Objective** | **Instruments** | **Key Findings** |
| --- | --- | --- | --- | --- | --- | --- |
| Brodersen et al, 2017. Denmark | Prospective Cohort | Screening | 433  M: 433 (100) | New COS-AAA questionnaire validation. | 1. Psychosocial consequences of being diagnosed with AAA during screening, it was based on Engel’s, the bio-psycho-social model. 2. Core-questionnaire COS (Consequence of Screening): Part I (anxiety, behavioral, sense of dejection, sleep, single item) and Part II (relaxed/calm, social relations, existential values, impulsivity, empathy) | This study showed that a condition-specific tool is an appropriate and valid way to assess psychosocial factors in AAA screening. |
| Duncan et al, 2021. United Kingdom | Prospective Cohort | Screening | 4613  F: 4613 (100) | Prevalence of AAA in women and Quality of life assessment. | 1. Quality of Life (QoL) was assessed using EQ-5D | The study observed lower quality-of-life scores after screening, suggesting that AAA screening in women may have negative effects; however, the absence of a comparable unscreened group limits this conclusion. |
| Endicott et al, 2016. United States | Prospective Cohort | Elective Repair | 9030  F: 46 (0.5)  M: 8,984 (99.5) | Mortality and functional status. | 1. An 11-point frailty index (FI) to demonstrate increased morbidity and morbidity in patients undergoing vascular procedures. 2. 3-point ordinal scales, were converted into binominal scale Normal (1= completely independent) and Abnormal (2 or 3= partially to totally dependent). | The study found that functional status is a quick, practical predictor of mortality in AAA patients and may help guide preoperative risk assessment in older adults needing repair. It remained strongly associated with mortality among patients over 80 years (P < .001). |
| Fattahi et al, 2024. Sweden | Retrospective Cohort | Surveillance | 1508  F: 809 (53.6)  M: 699 (46.4) | Awareness of heritability for AAA and possible anxiety in male and female adult offspring of AAA patients compared to matched controls. | 1. Hospital Anxiety and Depression Scale (HADS: HADS-A, HADS-D) 2. EQ-5D. 3. Health status measurement through a self-rating on a thermometer that ranges from 0-100. | Female adult offspring showed higher mean HADS and HADS-A scores, with more women exceeding the HADS-A threshold of 8 compared with men (17% vs. 11%, p = 0.025). Women also had higher EQ-5D index scores than men (6.8 ± 2.3 vs. 6.4 ± 2.0, p = 0.020). |
| Fenton et al, 2021. United Kingdom | Retrospective Cohort | Elective Repair | 471* | Effect of exercise programs on perioperative and postoperative morbidity and mortality.  Secondary: quality of life. | 1. Exercise therapy (circuit training, moderate-intensity continuous exercise and high-intensity interval training) 2. EQ-5D 3. EQ-VAS 4. SF-36 | The study found minimal to no differences between the exercise and usual-care groups in ICU stay duration, total hospital stay, or quality-of-life outcomes. |
| Janssen et al, 2020. The Netherlands | Prospective Cohort | Elective Repair | 265  F: 94 (35.0)  M: 171 (65.0) | Impact of elective major abdominal surgery and subsequent post-op delirium on Quality of Life, cognitive functioning and depressive symptoms. | 1. QoL was assessed with the World Health Organization Quality of Life-BREF questionnaire (WHOQOL-BREF). 2. Cognitive functioning was measured with the Mini-Mental State Examination. 3. Depressive symptoms with the CES-D 16. | Quality of life was assessed across four domains, physical health, psychological health, social relationships, and environment. Surgery did not impact the social relationships, environmental, or overall domains, and differences in physical and psychological scores between diagnostic groups disappeared after adjusting for baseline values. Physical and psychological QoL declined at discharge; however, physical health returned to baseline by 6 months and remained stable at 12 months. |
| Lee et al, 2017. United Kingdom | Prospective Cohort | Screening | 191  F: 17 (9.0)  M: 174 (91.0) | Preferences for different aspects of management in the hypothetical scenario of small or fast growing. | 1. Online original survey. | Participants most often prioritized research aimed at understanding why AAAs develop and discovering medications that could shrink or halt aneurysm growth. The study provides a snapshot of patient perspectives on key AAA research priorities. |
| Li et al. 2019. United States | Retrospective Cohort | Elective Repair | 177  F: 37 (18.0)  M: 140 (82.0) | To evaluate mortality and morbidity, but also high costs EVAR compared with open repair. | 1. EQ-5D-VAS  2. EQ-5D | ENGAGE PAS de novo patients had lower VAS scores at discharge and reported more issues with mobility, self-care, activity, and pain compared with baseline, but these changes resolved by the 1-month follow-up. |
| Manchin et al, 2024. United Kingdom | Prospective Cohort | Elective Repair | 98* | To develop an international core outcome set for intact AAA repair. | 1. SF-36 2. EQ-5D | The Delphi consensus identified key QoL outcomes including overall quality of life, pain, anxiety, work capacity, cognitive, physical, social, and sexual function, and discharge destination, along with patient satisfaction measures such as satisfaction with decision-making and treatment. |
| Nano et al, 2014. Italy | Retrospective cohort | Elective Repair | 118  F: 8 (6.7)  M: 100 (93.3) | To report retrospectively experience on post implantation syndrome after use of endograft in patients undergoing EVAR | 1. SF-12 health survey | QoL assessments showed that patients who developed post-implantation syndrome reported greater limitations in daily physical activities and higher levels of emotional distress, including depression and anxiety, compared with those without PIS. |
| Peach et al. 2016. United Kingdom | Retrospective Cohort | Surveillance and Elective Repair | 191  F: 18 (10.0)  M: 172 (90.0) | To evaluate Quality of Life, symptoms and treatment satisfaction. | 1. AneurysmDQoL 2. AneurysmSRQ 3. AneurysmTSQ | AneurysmDQoL trends showed that the surgical group experienced a growing negative impact on QoL over time, whereas the EVAR group showed improvement. EVAR patients reported increasing symptom bother with time, while those who underwent open repair reported decreasing bother. |
| Philips et al, 2021. United Kingdom | Prospective Cohort | Screening | ** | To identify patient reported outcome measure (PROM) to quantify anxiety in UK National Abdominal Aortic Aneurysm Screening Program. | 1. Psychological Consequences of Screening (PCQ) questionnaire. | Knowing they have an AAA may cause anxiety that negatively affects quality of life, potentially offsetting the benefits of screening and surveillance. |
| Suckow et al, 2016. United States | Retrospective cohort | Surveillance and elective repair | 1008  F: 252 (25.0)  M: 756 (75.0) | To evaluate and quantify AAA-specific knowledge in patients under AAA surveillance and in patients who have undergone AAA repair | 1. Original Survey: patient-reported most important source of information, patient-reported amount of information from doctor, and questions designed to assess abdominal aortic aneurysm-specific knowledge. | A national survey of AAA-specific knowledge showed that patients had limited understanding of their condition, which may lead to increased anxiety and uninformed decisions. |
| Tew et al, 2017. United Kingdom | Prospective Cohort | Elective Repair | 53  F: 3 (6.0)  M: 50 (94.0) | To assess the feasibility of a preoperative high-intensity interval training program in patients awaiting elective AAA repair. | 1. Physical Activity Enjoyment Scale. 2. Health-related quality of life (Short Form SF 36-item) and mental health MH subscales, EQ-5D and EQ-VAS. | Exercise may lead to modest improvements in SF-36 scores, but gains in overall quality of life (EQ-5D) and mental health were not statistically significant. |
| van der Veen et al, 2021. The Netherlands | Prospective Cohort | Elective Repair | 100  F: 3 (3.0)  M: 97 (97.0) | To evaluate the new device after 12 months. | 1. Walking Impairment Questionnaire (WIQ) 2. EQ-5D 3. International Index of Erectile Function 5 | Favorable clinical outcomes were observed at the 12-month follow-up. |
| van Schaik et al. 2020. The Netherlands | Prospective Cohort | Elective Repair | 101* | To evaluate vascular surgeons’ knowledge and appreciation of ejaculatory dysfunction after open aortic aneurysm repair | 1. Designed questionnaire based on a review of the literature: 9-item questionnaire addresses vascular surgical experience, knowledge of, and attitudes on postoperative ejaculation disorders, nerve-sparing exposure techniques and outpatient clinic-related practices. | Limited data exist on the incidence of ejaculation disorders following aortic reconstruction. Dutch vascular surgeons recognize these postoperative issues after infrarenal repair, but important gaps remain in understanding the underlying anatomy and pathophysiology. |

*Sex distribution not specified.

** Total population not specified.

**Abbreviations:** AAA: Abdominal Aortic Aneurysm; QoL: Quality of Life; COS-AAA: Consequences of Screening in Abdominal Aortic Aneurysm questionnaire; EQ-5D-EL: EuroQol 5-Dimension 5-level questionnaire; EQ-VAS: EuroQol Visual Analogue Scale; HADS-D: Hospital Anxiety and Depression Scale (HADS), depression domain; HADS-A: Hospital Anxiety and Depression Scale (HADS), anxiety domain; SF-36: Short Form 36 health survey; SF-36 MH: Short Form 36 health survey, Mental Health domain; SF-36 PF: Short Form 36 health survey, Physical Functioning domain; CES-D 16: Center for Epidemiologic Studies Depression 16 questionnaire; SF-12: 12-ITEM Short-Form health survey; PCS: Psychological Consequences Questionnaire; PACES-S: Physical Activity Enjoyment Scale; WIQ: Walking Impairment Questionnaire; IIEF: International Index of Erectile Function; WHOQOL-BREF: World Health Organization Quality of Life-Brief; AneurysmDQoL: Aneurysm-Dependent Quality of Life; AneurysmSRQ: Aneurysm-Symptom Rating Questionnaire; AneurysmTSQ: Aneurysm-Treatment Satisfaction Questionnaire; EVAR:Endovascular Aneurysm Repair; ENGAGE PAS: Endurant Stent Graft System Post Approval Study; IGQ: Informational Gain Questionnaire; PSQ: Patient Satisfaction Questionnaire; PIS: Post implantation syndrome; ICU: Intensive Care Unit; ADLs: Activity of Daily Living.

**Table S3.** Table of Excluded Studies with Reasons for Exclusion

| **Author** | **Year** | **Tittle** | **Exclusion Reason** |
| --- | --- | --- | --- |
| Anderson | 2020 | Patient information sources when facing repair of abdominal aortic aneurysm | Wrong Outcomes |
| Bains | 2021 | Screening Older Adult Men for Abdominal Aortic Aneurysm: A Scoping Review | Wrong Outcomes |
| Ball | 2016 | Screening individuals with intracranial aneurysms for abdominal aortic aneurysms is cost-effective based on estimated coprevalence | Wrong Patient Population |
| Bockler | 2020 | Improvements in patient outcomes with next generation endovascular aortic repair devices in the ENGAGE Global Registry and the EVAR-1 clinical trial | Full Text No Found |
| Boult | 2015 | Self-reported fitness of American Society of Anesthesiologists class 3 patients undergoing endovascular aneurysm repair predicts patient survival | Wrong Outcomes |
| Boult | 2017 | Fitness plus American Society of Anesthesiologists grade improve outcome prediction after endovascular aneurysm repair | Wrong Outcomes |
| Cherniavskii | 2018 | Exoprosthetic repair of the ascending portion of the aorta: midterm results | Full Text No Found  Wrong Language Abstract |
| Chun | 2016 | Surveillance outcomes of small abdominal aortic aneurysms identified from a large screening program | Wrong Outcomes |
| Dodds | 2023 | Characterizing recovery following abdominal aortic aneurysm repair using cardiopulmonary exercise testing and patient reported outcome measures | Full Text No Found |
| Glover | 2014 | Cost-effectiveness of the National Health Service Abdominal Aortic Aneurysm Screening Programme in England | Wrong Outcomes |
| Hager | 2017 | Revisiting the cost-effectiveness of screening 65-year-old men for abdominal aortic aneurysm based on data from an implemented screening programme | Missing Quality of Life Information |
| Hamel | 2018 | Potential benefits and harms of offering ultrasound surveillance to men aged 65 years and older with a subaneurysmal (2.5-2.9 cm) infrarenal aorta | Wrong Outcomes |
| Hatzl | 2023 | “Mixed Reality" in patient education prior to abdominal aortic aneurysm repair | Wrong Outcomes. Survey not validated. Excluded during full text review. |
| Inagaki | 2017 | Preoperative hypoalbuminemia is associated with poor clinical outcomes after open and endovascular abdominal aortic aneurysm repair | Wrong Outcomes |
| Jacomelli | 2012 | Editor's Choice - Inequalities in Abdominal Aortic Aneurysm Screening in England: Effects of Social Deprivation and Ethnicity | Wrong Outcomes |
| Knappich | 2024 | Endovascular aortic repair with sac embolization for the prevention of type II endoleaks (the EVAR-SE study): study protocol for a randomized controlled multicentre study in Germany | Study Protocol |
| Lindholt | 2021 | Clinical Benefit, Harm, and Cost Effectiveness of Screening Men for Peripheral Artery Disease: A Markov Model Based on the VIVA Trial | Wrong Patient Population |
| Matsumura | 2015 | Costs of repair of abdominal aortic aneurysm with different devices in a multicenter randomized trial | Wrong Outcomes |
| Nair | 2019 | Health gains, costs and cost-effectiveness of a population-based screening programme for abdominal aortic aneurysms | Wrong Outcomes |
| Powell | 2015 | Emerging strategies to treat ruptured abdominal aortic aneurysms | Missing Quality of Life Information |
| Reile | 2020 | The Cost-Effectiveness of Abdominal Aortic Aneurysm Screening in Estonia | Wrong Outcomes |
| Sogaard | 2018 | Cost-effectiveness of population-based vascular disease screening and intervention in men from the Viborg Vascular (VIVA) trial | Missing Quality of Life Information |
| Sweeting | 2018 | Analysis of clinical benefit, harms, and cost-effectiveness of screening women for abdominal aortic aneurysm | Wrong Outcomes |
| Sweeting | 2021 | Evaluating the Cost-Effectiveness of Changes to the Surveillance Intervals in the UK Abdominal Aortic Aneurysm Screening Programme | Wrong Outcomes |
| Takeuchi | 2019 | Using bifurcated endoprosthesis after iliac artery recanalization for concomitant abdominal aortic aneurysm and chronic total occlusions of access routes | Wrong Outcomes |
| Torsello | 2016 | Ultrasound screening for abdominal aortic aneurysms - a rational measure to prevent sudden rupture | Non-English Article |
| Tyrovolas | 2022 | Global, regional, and national burden of aortic aneurysm, 1990-2017: a systematic analysis of the Global Burden of Disease Study 2017 | Wrong Outcomes |
| Ultee | 2015 | Low Socioeconomic Status is an Independent Risk Factor for Survival After Abdominal Aortic Aneurysm Repair and Open Surgery for Peripheral Artery Disease | Wrong Patient Population |
| Van den Berg | 2024 | Protocol for an independent patient data meta-analysis of prophylactic mesh placement for incisional hernia prevention after abdominal aortic aneurysm surgery: a collaborative European Hernia Society project (I-PREVENT-AAA) | Wrong Outcomes |
| Van Leeuwen | 2024 | Health Literacy and Disease Knowledge of Patients With Peripheral Arterial Disease or Abdominal Aortic Aneurysm: A Scoping Review | Full Text No Found |
| Von Allmen | 2019 | Randomized controlled comparison of cross-sectional survey approaches to optimize follow-up completeness in clinical studies | Missing Quality of Life Information |
| Wanhainen | 2016 | Outcome of the Swedish Nationwide Abdominal Aortic Aneurysm Screening Program | Wrong Outcomes |
| Wickramasekera | 2019 | Strength of public preferences for endovascular or open aortic aneurysm repair | Wrong Outcomes |
| Ying | 2019 | Abdominal Aortic Aneurysm Screening: A Systematic Review and Meta-analysis of Efficacy and Cost | Wrong Outcomes |
| Zarrouk | 2016 | Cost-effectiveness of Screening for Abdominal Aortic Aneurysm in Combination with Medical Intervention in Patients with Small Aneurysms | Wrong Outcomes |

**Table S4.** Evaluation of Psychometric Properties of Patient-Reported Outcome (PRO) Instruments in Clinical Trials based on the U.S. Department of Health Guidance (19).

| **Psychometric Property** | **Domain** | **Definition** | **Review Considerations** |
| --- | --- | --- | --- |
| Reliability | Test-retest | Consistency of scores over time when the measured concept remains unchanged | - Pearson’s correlation - Time period of assessment - In Peach et al 2026, the Intraclass correlation coefficients were guided by: excellent (≥ 0.81), good (0.61–0.80), moderate (0.41–0.60), poor (≤ 0.40). |
|  | Internal consistency | Extent to which items comprising a scale measure the same concept. Intercorrelation of items that contribute to a score. | - Cronbach’s alpha for summary scores - Item-total correlations |
|  | Inter-interviewer reproducibility | Agreement between responses when administered by multiple interviewers | - Interclass correlation coefficient (ICC) |
| Validity | Content-related | Evidence that the instrument measures the domains or concept of interest. | - Derivation of all items - Qualitative interview schedule - Interview or focus group transcripts - Items derived from transcripts - composition of patients used to develop content - cognitive interview transcripts to evaluate patient understanding |
|  | Construct-related validity | Evidence that alignment of items, domains, and concepts with the PRO instrument’s framework and validation hypotheses | - Strength of correlation testing a priori hypotheses. - Ability of the PRO instrument to differentiate between groups that were hypothesized to differ |
|  | Predictive validity | Prediction of future events or status based on changes in PRO scores | - Accuracy of PRO scores in predicting subsequent events or outcomes |
| Ability to detect change | Effect size and standard error of measurement among others | PRO scores remain stable with no patient change and shift as predicted with notable patient change, measured by effect size, and are specific to the time interval | - Ability to detect change demonstrated in a comparative trial, comparing mean group scores or patient response proportions, and ability to detect change assessed for the appropriate study time interval. |
| Interpretability | Minimum important difference (MID), the smallest clinically important difference | Difference in mean score between treatment groups that provides evidence of treatment benefit, using various approaches such as distribution-based, clinical or nonclinical anchor, or combination of approaches | - Feedback on the review of MID derivation and application in clinical trials |
|  | Responder definition | Score change indicating clear evidence of treatment benefit, based on distribution-based, clinical anchor, empirical rule, or combined approaches | - Feedback on the review of responder definition derivation and application in clinical trials |

**Abbreviations***: MID*, Minimum important difference; *PRO,* patient-reported outcome; *ICC,* interclass correlation coefficient.

**Table S5.** Overview of patient-reported outcome measure (PROM) domains, psychometric properties, and expansion of the evidence base

| **Instrument** | **Conceptual framework/domain** | **Reliability** | | | **Validity** | | | **Ability to detect change** | **Interpretability** | |
| --- | --- | --- | --- | --- | --- | --- | --- | --- | --- | --- |
|  |  | **Test-retest** | **Internal consistency** | **Inter-interviewer reproducibility** | **Content validity** | **Construct validity** | **Predictive validity** | **Effect size and standard error** | **Minimum important difference** | **Responder definition** |
| **Disease-Specific Health Questionnaire** | | | | | | | | | | |
| AneurysmDQoL | 22 domain-specific items: leisure, work, long distance journeys, holidays, do physically, family life, friendships and social life, closes personal relationships, sex life, getting out and about, household tasks, do things for others, enjoy food, feelings about the future, finance, having to depend on others, health, others fuss or worry, energy, physical discomfort, anxiety, think clearly/concentrate and remember | ICC = 0.66 | 0.959 | Not reported | Nine focus groups were interviewed and developed a comprehensive and relevant key life domain affected by AAA | r > 0.438 | Lower score is associated with higher post-operative complications, poorer recovery and reduced long-term survival | Not reported | MID: changes 5-10 points, may indicate a clinically meaningful improvement | Patient experienced clinically significant improvement |
| AneurysmSRQ | 44 items with six domains were examined: emotion, appetite, lower limb, cognitive, general malaise and gastrointestinal | ICC = 0.81 | 0.906 | Not reported | Nine focus groups were interviewed and developed a comprehensive and relevant symptoms experienced in patients affected by AAA | r > 0.4 | Higher scores may predict increased post-operative complications, and lower health related quality of life | Not reported | MID: changes 2-4 points, may indicate improvement or worsening | Patient experienced clinically significant improvement |
| AneurysmTSQ | 11 items, measuring total treatment satisfaction for patients who have undergone AAA repair | ICC = 0.88 | 0.902 | Not reported | It was established through patient and clinician involvement | r > 0.476 | High satisfaction scores are linked to better adherence and care | Not reported | MID: changes 5-10% in satisfaction may indicate a meaningful difference | Patient experienced clinically significant improvement |
| COS-AAA | This instrument consists of two parts: part I encompasses 18 domains with more than 70 items and part II encompasses 5 domains including 21 items | ICC = 0.70 – 0.88 | Internal consistency was confirmed using Rasch modeling. 51 items fitting the model. Value not specified | Not reported | High content validity through qualitative interviews with men under surveillance for screening-detected AAA, ensuring the inclusion of relevant psychosocial items | Not reported | Not reported | Not reported | Not reported | Not reported |
| Aortic Aneurysm QoL Survey | 2 sub-surveys: post repair 55 questions, and living with AAA 62 questions, with 2 domains: emotional impact and behavioral changes | ICC = 0.91 | High internal consistency, with an ICC of 0.91, consistency in measuring the same construct | Not reported | Covering various psychosocial domains relevant to the target population, identifying important elements of AAA-specific knowledge | Not reported | Not reported | Not reported | Not reported | Not reported |
| **Generic-Specific Health Questionnaire** | | | | | | | | | | |
| EQ-5D-5L | 5 domains, each with one item: mobility, self-care, usual activity, pain/discomfort, and anxiety/depression | ICC = 0.77 | 0.70-0.90 | Consistency of responses when different interviewers administer the same instrument | This instrument was developed through a comprehensive process, including expert input, covering essential health dimensions and it has been adapted and tested in multiple languages and cultural settings | It is sensitive to changes in health status, demonstrating it accurately reflects the underlying construct of health-related quality of life | Demonstrated by its ability to predict healthcare costs and treatment outcomes, making it useful in clinical and research settings | Effect size of 0.5 or greater. Standard error is generally low | MID = 0.03-0.08 | Based on the MID, responder is classified whether their health status has changed by a meaningful amount |
| 5Q-VAS | 5 domains, each of them with a range of 0-100 self-reported value: physical health, mental health, social functioning, self-perception, and general health | Same as above | Same as above | Same as above | Same as above | Same as above | Same as above | Same as above | Same as above | Same as above |
| SF-36 | 36 items across 8 domains: physical functioning, role physical, bodily pain, general health, vitality, social functioning, role emotional, and mental health | Good consistency over time ICC = > 0.80 | 0.70-0.90 | Different interviewers can administrate the tool with similar results | This instrument captures the broad aspect of health-related quality of life. content experts and patient groups were involved in the development, including relevant items | Strong construct validity. Factor analyses confirm that the eight domains represent distinct but related component of health | It can predict health-related outcomes, including future health status, healthcare utilization, and work productivity | Effect size: 0.5-0.8 medium to large | MID in physical functioning change of 5 to 10 points.  MID in mental health change of 3 to 5 points | Based on the MID, identifies patients with a clinically significant change in health |
| PCQ | 6 domains with 24 items: anxiety, stress, coping, perceived risk, reassurance, and regret | Not assessed | Very good | Not reported | Insufficient data for content validity | Inadequate | Not reported | Not reported | Not reported | Hypothesis testing before and after intervention: very good. |
| PACES-S | 18 items with 4 domains: positive affect, intrinsic motivation, engagement & flow, and lack of negative feelings | ICC = 0.80 – 0.90 | 0.85-0.96 | Not reported | Experts in exercise psychology have reviewed the scale to ensure all relevant dimensions of enjoyment are covered | PACES has been related to constructs like motivation, adherence to exercise programs, and intrinsic motivation, supporting its construct validity | Enjoyment plays a crucial role in sustaining an active lifestyle | Not reported | Not reported | Not reported |
| WIQ | 3 domains with 24 items: walking distance, walking speed, and stair climbing | ICC = 0.74, good | 0.90. high internal consistency | High reliability across different administration methods | Expert input and patient-reported difficulties in walking, ensuring it captures key aspects of walking impairment. | WIQ scores correlate significantly with objective measures of walking ability | It has been used to predict mobility decline and the effectiveness of interventions such as exercise therapy | Moderate to large effect size has been reported | Changes of 5 to 10 points in WIS score are considered clinically meaningful | Increase in WIQ score beyond the MID or significant improvements in walking tests |
| IIEF | 15 items covering 5 domains: erectile dysfunction, orgasmic function, sexual desire, intercourse satisfaction, and overall satisfaction. | ICC = 0.82-0.98 | ≥ 0.9 strong internal consistency | Not reported | Developed through expert consensus | Correlates strongly with self-reported sexual function | Can predict treatment outcomes and correlate with improvements in erectile dysfunction after interventions | Not reported | Changes of 4-7 points in erectile dysfunction domain is considered clinically meaningful | Patient experienced clinically significant improvement |
| WHOQOL-BREF | 26 items covering 4 domains: physical health, psychological health, social relationships, and environment (finances, security, safety, home environment, etc.) | ICC = 0.66 – 0.91 | High, 0.70-0.90 | ICC = 0.85 – 0.95 | Use of a broad, international expert review, based on diverse cultural and population data | The four domains are distinct and correlate well with other health-related quality of life measure | Higher scores have been associated with better health outcomes, including fewer hospital admissions and better overall health | Effect size between 0.2 to 0.5. standard error low, indicting good precision of the scores | MID: 5 to 10 points on the scale | Clinically meaningful change in at least one of the domains |
| **Psychopathology Screening** | | | | | | | | | | |
| HADS  (HADS-A, HADS-D) | 14 items divided into 2 domains: anxiety subscales and depression subscales | Not reporter | High 0.68-0.93 | Not reported | It focusses on psychological and cognitive aspects | Confirmatory factor analyses have generally supported the aspects of this instrument | Identifying individuals at risk for developing anxiety and depression disorders | Not reported | MID: 1.5 to 2 points on each subscale | Not reported |
| CES-D 16 | 16 items grouped into 4 domains: depressed affect, positive affect, somatic symptoms, and interpersonal problems. | ICC = 0.70 | 0.80-0.90 | Strong agreement between different interviewers administering this instrument | Developed based on symptoms commonly associated with major depression | Four-domain structure it is validated in measuring depressive symptoms | Scores correlate with clinical depression diagnoses | Effect size = 0.50-0.80, moderate to large. Standard error low, meaning precise scoring | MID: 3-5 points, to be considered clinically significant | It is defined as improvement of MID |

**Abbreviations:** *AAA:* Abdominal Aortic Aneurysm; *COS-AAA*: Consequences of Screening in Abdominal Aortic Aneurysm questionnaire; *EQ-5D-EL:* EuroQol 5-Dimension 5-level questionnaire; *EQ-VAS:* EuroQol Visual Analogue Scale; *HADS-D:* Hospital Anxiety and Depression Scale (HADS), depression domain; *HADS-A*: Hospital Anxiety and Depression Scale (HADS), anxiety domain; *SF-36:* Short Form 36 health survey; *CES-D 16:* Center for Epidemiologic Studies Depression 16 questionnaire; *SF-12:* 12-ITEM Short-Form health survey; *PCQ:* Psychological Consequences Questionnaire; *PACES-S:* Physical Activity Enjoyment Scale; *WIQ*: Walking Impairment Questionnaire; *IIEF:* International Index of Erectile Function; *WHOQOL-BREF:* World Health Organization Quality of Life-Brief; *AneurysmDQoL*: Aneurysm-Dependent Quality of Life; *AneurysmSRQ:* Aneurysm-Symptom Rating Questionnaire; *AneurysmTSQ:* Aneurysm-Treatment Satisfaction Questionnaire; *EIS:* Emotional Impact Score.
